# Identification of Milk Component in Ancient Food Residue by Proteomics

**DOI:** 10.1371/journal.pone.0037053

**Published:** 2012-05-16

**Authors:** Chuan Hong, Hongen Jiang, Enguo Lü, Yunfei Wu, Lihai Guo, Yongming Xie, Changsui Wang, Yimin Yang

**Affiliations:** 1 Laboratory of Human Evolution, Institute of Vertebrate Paleontology and Paleoanthropology, CAS, Beijing, China; 2 Department of Scientific History and Archaeometry, Graduate University, Beijing, China; 3 Xinjiang Institute of Archaeology, Xinjiang Uygur Autonomous Region, Urumchi, China; 4 College of Life Science, Graduate University, CAS, Beijing, China; 5 Asia Pacific Application Support Center, AB SCIEX, Shanghai, China; University College London, United Kingdom

## Abstract

**Background:**

Proteomic approaches based on mass spectrometry have been recently used in archaeological and art researches, generating promising results for protein identification. Little information is known about eastward spread and eastern limits of prehistoric milking in eastern Eurasia.

**Methodology/Principal Finding:**

In this paper, an ancient visible food remain from Subeixi Cemeteries (cal. 500 to 300 years BC) of the Turpan Basin in Xinjiang, China, preliminarily determined containing 0.432 mg/kg cattle casein with ELISA, was analyzed by using an improved method based on liquid chromatography (LC) coupled with MALDI-TOF/TOF-MS to further identify protein origin. The specific sequence of bovine casein and the homology sequence of goat/sheep casein were identified.

**Conclusions/Significance:**

The existence of milk component in ancient food implies goat/sheep and cattle milking in ancient Subeixi region, the furthest eastern location of prehistoric milking in the Old World up to date. It is envisioned that this work provides a new approach for ancient residue analysis and other archaeometry field.

## Introduction

Along with the development of civilization, domesticated animals, such as cattle, goat, sheep and horse, became more and more important in farming practices. Series of reports have indicated that these animals were initially tamed for the production of meat, hide and bone, and later for a more diversified exploitation of their lifetime products and uses, including milk, wool, as well as riding, transportation or tillage [1]; and this process was entitled “Secondary Products Revolution (SPR)” by Andrew Sherratt [2], [3]. Among these secondary uses of livestock, dairying played a significant role in the agrarian civilization process of Europe, and also was crucial in the emergence and evolution of the nomadic culture of the Eurasian grasslands, due to the special nutritional value of dairy products [4], [5]. In consideration of this, much research focused on ancient dairying in order to provide more evidence about the progress and spread of animal domestication [6], [7].

The major approaches applied in research on dairying are as follows: (1) Lipid analysis, *e.g.*, the analysis of lipids preserved in pottery, which indicated that the exploitation of milk could be shifted to an earlier date, about in 7,000 BC [8]; (2) Immunological method, *e.g.*, Enzyme Linked Immunosorbent Assay (ELISA), applied in the identification of the presence of casein preserved in artisan materials [9]; (3) Zooarchaeological method, especially the harvest profile (age-at-death) of unearthed animal skeletons, *e.g.*, the quantity and the distribution of the age of slaughtered cattle unearthed in the Harappa relics in Pakistan showed that the mature cow had been raised for a longer time, which might have been used for the production of milk [10]; (4) The distribution of lactase persistence genes, *e.g.*, the ability of lactase persistence possibly emerged from people who had lived in the west of the Urals and the north of the Caucasus [11]. (5) Determining the weaning time of calves through the detection of nitrogen isotope [12].

However, in spite of the fact that cattle, sheep and goat can offer products with similar nutritional properties, the economic, social and cultural implications of using these animals for these purposes were very different. Determining the species of animal exploited for milk is also vital to understanding dairying [13]. Furthermore, zooarchaeological harvest profiles are the most useful method to observe general shifts of animal exploitation, but not to identify specific products [14]. Thus, the identification of the animal species related to milk residues has become a hotspot in international academic research. Proteomic methods have been recently applied in archeology and arts, thus opening a new window for residue analysis [15]–[23]. Proteomic approaches based on mass spectrometry have been particularly used to clearly discriminate sheep and goat from bovine alpha casein used in mural painting [18]. Milk additives (bovine milk, curd, and whey) in historical building materials have been identified via proteomic analysis and several sequences have been confirmed “*de novo*” by mass spectrometry [19]. These works imply that the proteomic method could be subsequently used to identify milk component in food residue and determine the precise origin of milk, which would help better understand prehistoric animal exploitation and subsistence shift according to SPR theory.

**Figure 1:**
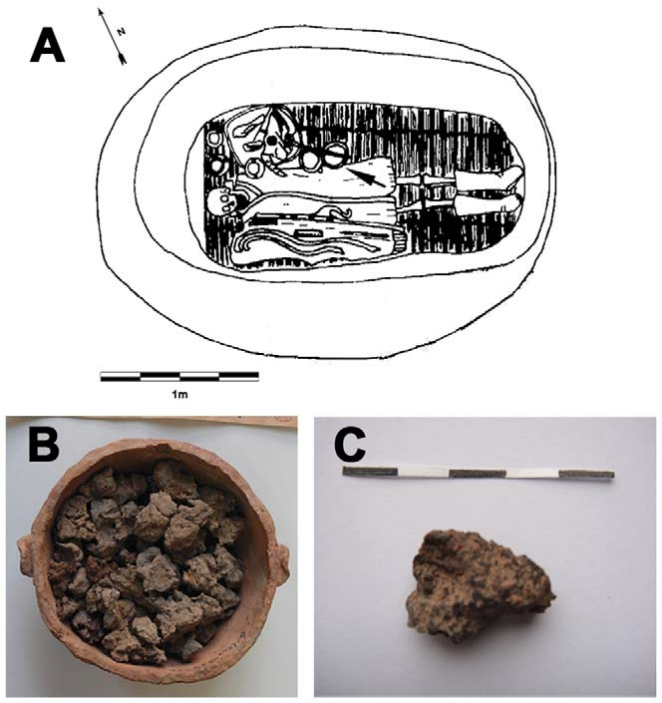
Samples chosen for archaeological analysis. A) The line drawing of tomb where ancient residue existed. The bottom layer of III M27, showing the black food residues (arrow) in the pottery bowl (M27:9, adapted from XIA & MT, 2002); B) black residues in the pottery bowl; C) residues chosen for analysis. Scale bar = 5 cm (photo b supplied by Prof. Enguo Lü).

The spread of domesticated cattle, sheep, and goats into ancient China came at least 5000, 4000, and 3700 years ago, respectively [24]; but there is little information about the spread of secondary products exploitation in eastern Asia, which has been largely ignored since SPR theory was suggested [25]. In the present study, a monoclonal ELISA technique has been used for the preliminary identification of cattle casein contained in an ancient black food residue. The positive result of ELISA analysis showed that the cattle casein concentration in the ancient food was 0.432 mg/kg, a little higher than the detection limit (0.12 mg/kg) and lower than the limit of quantification (0.5 mg/kg) [26]. Given this lower cattle casein concentration, in order to claim whether ELISA result is reliable and obtain more information on dairying, an improved analysis strategy was adopted by using LC-MALDI-TOF/TOF-MS.

## Materials and Methods

### Archaeological Samples

Ancient black food residues were taken from a pottery bowl (coded as No. M27: 9) in the tomb M27 (see Figure 1) at the Subeixi site located in the Turpan Basin, Shanshan County, Xinjiang Uighur Autonomous Region, China. The Turpan District is famous for its representative continental desert climate, with annual precipitation of 25.2 mm and an evaporation rate as high as 2500 mm. Site description and archaeological background were previously introduced in detail in other sources [27]–[29]. In brief, the food residues obtained are believed to have existed in cal. 500-300 BC. The tomb M27 in No.3 cemetery is an earth-pit burial consisting of three layers. The skeletons of an adult male, a female, and an infant were found in the upper layer; the skeleton of another adult female was buried in the middle layer; an elderly man was interred on the lower layer, and the black food residue which was analyzed was found in a pottery bowl to the left side of his waist.

**Table 1:** Results of MS/MS by LC-MALDI-TOF.

No.	Sequence	Protein	Species
A	FVVAPFPEVFR	α_S1_-casein	sheep and goat
B	YLGYLEQLLR	α_S1_-casein	sheep, and bovine
C	FFVAPFPEVFGK	α_S1_-casein	bovine
D	DMPIQAFLLYQEPVLGPVR	β-casein	bovine, sheep, goat, water buffalo

### Protein extraction

The black food residue was firstly ground by hand with an agate mortar. Next, 0.1 g of ancient powder was extracted with 1 mL PBS at room temperature for 20 min. The supernatant was then collected and placed into a centrifuge vial (1.5 mL) without protein pollution, and clarified by centrifuging (2500 g for 10 min). Finally, protein extracts were diluted 10 times for later analysis.

### Tryptic digestion

1 mL of protein extract was diluted with the addition of 20 µL of 50 mM DTT at 56°C for 30 min. The sample was then allowed to re-equilibrate to room temperature, alkylated with the addition of 20 µL of 100 mM iodoacetamide, and then incubated for 1 hour at 37°C under slight vortex. Trypsin was subsequently added in a 1∶30 (trypsin∶sample) ratio twice: once at the start and once after 2 hours. Finally, digestion was completed overnight at 37°C.

**Figure 2:**
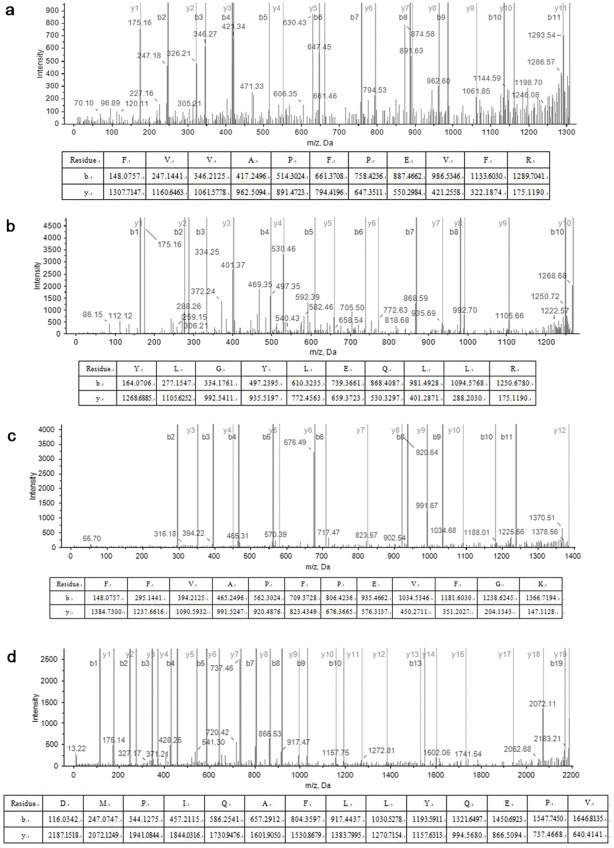
Mass spectra of four identified sequences.

**Figure 3:**
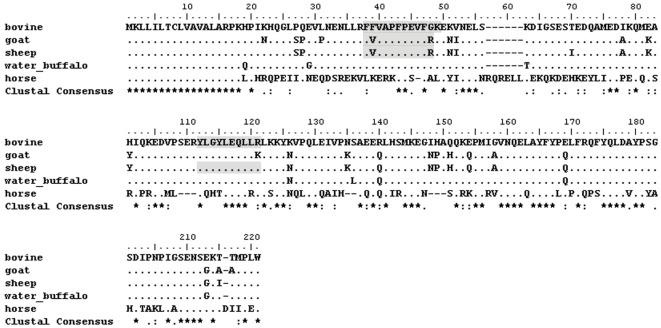
Multiple sequence alignment. Multiple sequence alignment of alpha S1 casein from bovine (Accession: ACG63494.1 GI: 195973860), goat (Accession: CAA51022.1 GI: 311943), sheep (Accession: ACJ46472.1 GI: 213391435), water buffalo (Accession: AAX49507.1 GI: 61658244) and horse (Accession: NP_001075352.1 GI: 126352302). The detected peptides by LC-MALDI-TOF-MS/MS are light grey. “*” identical residues, “:” conserved residues, and “.” semi-conserved residues.

### Reversed-Phase Separation of Peptides

Peptides separation was performed on Tempo™ LC-MALDI spotting system (AB SCIEX, Foster City, CA). 10 µL of digested samples was injected and captured on a 0.3×10 mm ProteCol™ trap column (3 µm, C18, SGE Analytical Science, AU) and then eluted and separated from a trap column hooked in series with a 0.2×150 mm analytical column (3 µm, C18, Michrom Bioresource, Auburn, CA). Peptides were resolved by dual solvent gradient elution (A: 2% ACN, 0.1% TFA; B: 98% ACN, 0.1% TFA) of 5–35% B in 30 min, 35–90% B in 5 min at a flow rate of 2 µL/min. The column effluent was monitored at 214 nm using a 3-nL UV flow cell, and after UV detection, was mixed with the MALDI matrix in a 1∶1 ratio through external syringe pump and spotted onto a MALDI plate in a 44×28 array at 8 s intervals. The matrix solution was 5 mg/mL α-cyano-4 hydroxycinnamic acid dissolved in 70∶30 ACN-H_2_O.

**Table 2:** The concentration of standard bovine casein and sample's OD absorbance.

No.	Solution added	Concentration of bovine casein (mg/kg)	OD value(450 nm)
**1**	Dilution buffer	0	0.073
**2**	bovine casein Standard 1	0.5	0.218
**3**	bovine casein Standard 2	1.5	0.366
**4**	bovine casein Standard 3	4.5	0.735
**5**	bovine casein Standard 4	13.5	1.507
**6**	ancient sample	0.432	0.180

**Figure 4:**
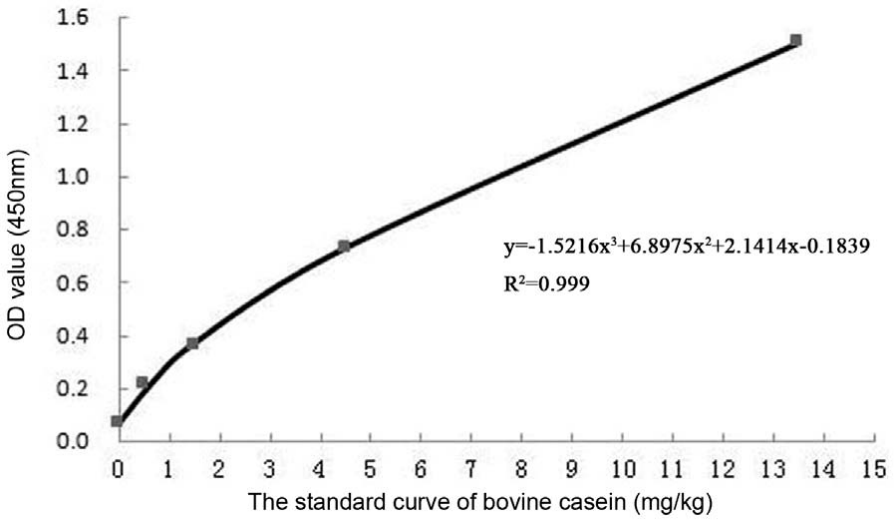
The standard curve of bovine casein. According to the ELISA procedures and the manual of the kit (RIDASCREEN® FAST Casein (Art. No.: R4602), R-Biopharm AG, Darmstadt, Germany), about 0.1 g ancient sample was taken to do the test. The standard samples and the ancient sample's OD values can be seen in Table 2. Based on the data illustrated in Table 2, the bovine casein concentration is 0.432 mg/kg, as the standard curve showed.

### MS/MS *analysis by* MALDI-TOF/TOF

Mass spectrometric analysis was performed on a 4800 MALDI-TOF/TOF Analyzer (AB SCIEX, Foster City, CA). MS-mode acquisitions consisted of 500 laser shots averaged from 10 sample positions (“search pattern positions”). After completing the MS analysis of an entire sample plate, precursors for MS/MS were selected by the instrument software. The software used signal-to-noise and mass tolerance thresholds to create a peak list for each 8-s RPHPLC fraction. Masses that recurred in several adjacent fractions were selected only from the fraction containing the peak apex based on intensity. The signal intensity for any given mass was required to be below the detection threshold for two fractions before that mass was eligible for reselection. The software was set to permit up to 30 MS/MS precursors to be selected for every 8-sec RPHPLC fraction. A mass range of 800 to 4000 Da. was considered for precursor selection. In the MS/MS acquisitions, 3000 laser shots were averaged from 60 sample positions. Two-keV MS/MS acquisition method (CID off) was used with a source voltage of 8 kV, a collision cell voltage of 6 kV, and a second accelerating voltage of 15 kV.

### Data analysis

Database searching was performed with the ProteinPilot3.0 software (AB SCIEX, Foster City, CA) utilizing Paragon™ as the search engine. Each MS/MS spectrum was searched against a database of all species (SwissProt, released March 2009). The search parameters allowed for cysteine modification by iodoacetamide and biological modifications programmed in the algorithm (*i.e.*, phosphorylations, amidations, semitryptic fragments, etc.). The detected protein threshold (unused protscore (conf)) in the software was set to 1.3 to achieve 95% confidence level, and identified proteins were grouped by the Pro Group algorithm (AB SCIEX) to minimize redundancy.

## Results

At the onset, the digested protein extract was directly analyzed through MALDI-TOF/TOF-MS, but no specific peptide peak was obtained. In this case, another analytical strategy, which separated sample peptides first by means of a Tempo LC-MALDI spotting system, was applied to avoid other laborious procedures for the removal of impurities and greatly increased the purity of the peptides.

Four peptide sequences related to casein were identified in the ancient food residue and are listed in Table 1; their respective mass spectra are reported in Figure 2. Sequence No.a is a homology sequence of alpha S1 casein 38–48 of sheep (*Ovis aries*) and goat *(Capra hircus)*; sequence No.b is a homology sequence of alpha S1 casein 106–115 of sheep and cattle (*Bos taurus*); No.d is a homology sequence of beta-casein of sheep, goat, cattle and water buffalo (*Bubalus bubalis*). Of note, sequence No.c is a conserved amino acid sequence assigned as unique peptide of alpha S1 casein 38–49 of cattle. These four sequences are marked by shading as showed in Figure 3.

In previous ELISA analysis, about 0.1 g of ancient sample was used [26]. The optical density (OD) values of cattle casein standards and the ancient sample were obtained in terms of the instruction manual of ELISA test kit (RIDASCREEN® FAST Casein (Art. No.: R4602), R-Biopharm AG, Darmstadt, Germany). Based on the concentrations and OD values of cattle casein standards in Table 2, the standard curve was fitted in Figure 4 to compute the bovine casein concentration of the ancient sample.

The antibodies in this ELISA kit specifically detect α-, β- and κ-caseins of cow and have no cross-reactivity to casein from sheep and goat. The proteomic identified unique peptide of cattle alpha S1 casein, which confirmed the qualitative ELISA result.

## Discussion

The identified alpha S1 casein and beta-casein from cattle, sheep and goat were characteristic of milk. Given the low concentration of cattle casein, these foods, therefore, may have been once dipped in milk. Furthermore, the identification of the unique peptide of cattle alpha S1 casein and the homology sequence from sheep and goat demonstrated that cattle and sheep/goat milking activity in Subeixi region existed at least 2300 years ago, thus permitting speculation that goat/sheep and bovine were raised by ancient people for milk during that period. Therefore, ancient people could get more animal protein without slaughtering animals, thus helping them better adapt to the hostile desert environment.

In Subeixi cemeteries, sheep/goat bones were buried, but the presence of bovine bones in this site is inconclusive [27], [28]; the present work not only reflects sheep/goat exploitation, but also supports the presence of cattle, and can be considered as evidence of cattle raising. According to ancient literature and archaeological findings (houses, pottery, grinding stones, crops, noodles and cakes made of millet dough, bows, bridles, leather, wool clothing, sheep remains etc.), the inhabitants of Subeixi site had a semi-agricultural and semi-pastoral (stock raising) life style [29]; and this work, which implies milking activities, further demonstrates the nature of the pastoral life of ancient people.

As late as AD 1500, the Old World could be divided into regions of milking and non-milking, and one major area of non-milking was located in eastern Asia, which included Southeast Asia and most of the Far East. The boundary of milking and non-milking people in China was roughly between the areas of Tibet and Mongolia, and eastern China [30], [31]. In addition, the distribution of lactose malabsorption is reflected in the spread of milking in these areas [32]. Studies regarding the origin and spread of dairying in the Near East and Europe have made great progress [25]; however, the timing of the emergence of dairying and its spread across Eurasian steppe to East Asia still remains unclear. To date, the Subeixi site is the furthest eastern location in the Old World that prehistoric milking has been detected, and the present work provides BP2300 as the latest time for the eastern spread of milking into the West of China. During this period, milking was not part of the traditional means of subsistence in Chinese agricultural areas, including the Gansu corridor, located in the east of Turpan basin. Thus, this work lends support to the eastern limits of prehistoric milking in western China.

Among residue analysis methods for dairying research, lipid analysis, using isotope of individual fatty acids, played an important role; this method has been widely applied to demonstrate the presence of milk fats [6], [8], [13], [33]–[39] and has further revealed the events of horse domestication [7]. However, certain deficiencies in this method not being able to distinguish the milk fat of cattle, sheep, and goat still exist. Although immunological analysis is very specific for certain species, only cattle casein could be detected and identified until now. In contrast to lipid and immunological analysis, the proteomic method is able to identify a more precise origin of protein in one analysis. For example, a multiple alignment of alpha S1 casein (see Figure 3) from sheep, goat, cattle, water buffalo, and horse was performed through Bioedit software. Figure 3 clearly shows that these animals, possibly raised for milk use, have distinct differences in amino acid loci of alpha S1 casein.

This work also shows that the cattle casein with concentration as low as 0.432 mg/kg in ancient samples could be detected and identified by proteomic methods. In fact, the concentration of cattle casein absorbed in the pottery body may be as low as 0.3–0.5 mg/kg [40]. Although there are some difficulties in direct extraction of protein from pottery shards, some successful extraction methods have been developed [41], [42]. Therefore, when using these same methods, it would be possible to use proteomic methods to detect and identify milk protein absorbed in pottery. As a result, proteomic methods would seem to have great potential in dairy research related to the origin and spread of domesticated animals.

### Conclusions

Although the focus on analysis of protein sequence has been one of regular approaches in modern biosciences for the past several years, similar methods have not been widely applied in ancient residue research. The present work proves the capability of LC-MALDI-TOF-MS/MS to identify the milk component preserved in ancient food remains, and clearly discerns the origin as well. Additionally, it is suggested that alpha S1 casein could be used for discrimination of the origin of milk in ancient residue. Moreover, considering both of the lower detection limit and higher accuracy, those involved in this study believe that not only the visible residue, but also the invisible residue, such as the protein absorbed in pottery, could be identified by proteomics methods. Furthermore, the results imply that animals such as goat/sheep and cattle were raised for milk, which enriches the understanding of Subeixi culture (cal. 500-300 BC), the furthest eastern location of prehistoric milking up to date.
